# Annotations

**Published:** 1897-12-25

**Authors:** 


### ANNOTATIONS.

### The Projectiles Used in the Græco-Turkish War.

IN a paper recently read before the West London Medico-Chirurgical Society, Mr. Davis, one of the surgeons who went out to the seat of war in Greece, gave some interesting details as to the nature of the wounds produced by the different weapons used, not only in that war, but in other parts of the world. The bullets extracted proved to be mostly Martinis. They never found a single Mauser bullet, but many soldiers had perforating wounds inflicted by this arm, and yet were not incapacitated; and it is reasonable to suppose that, from the great velocity of the Mauser bullet, it traverses the part struck, including perhaps the bone, and does but little damage compared with the Martini bullet, which, when it strikes a bone, fissures and splinters it in all directions. Several instances were met with in the Greek hospitals of wounds caused by fragments of shells. Shrapnel, however, did very little harm, which was explained by the fact that, whereas the impetus of the fragments of an exploded shell is largely derived from the charge of the shell itself, that of the shrapnel bullets is wholly derived from the velocity with which the case is travelling at the time when it explodes, and, therefore, the greater the distance from the gun the less damage the bullets do. "The Greek infantry preferred not to expose themselves to the Turkish shell firing, or indeed to any other for the matter of that, and hence their comparative immunity." The Turks, however, seemed to have suffered pretty severely from the shrapnel fire of the Greeks.

### Expanding Bullets.

THE Le Bel bullets used by the French, like the Mauser, and like the first Lee-Metfords, consist of a soft lead core surrounded by a nickel casing. Theoretically this form of bullet flattens or splashes on impact, but in practice this is found not always to happen, and in the Chitral expedition the Lee-Metford bullets were found not to be effective in stopping "rushes." By the simple device, however, of cutting through the nose of the bullet, or of rubbing it against a stone, and thus exposing the soft lead core, the bullet was made to expand on impact, and thus to produce the desired effect by immediately incapacitating those who were wounded by it. The small amount of immediate effect produced by the Lee-Metford bullet has been proved experimentally. "Three shots were fired, not by me," says Mr. Davis, "at an old donkey who was eating hay in a paddock. The bullets passed 'clean through' him, but he went on eating as though nothing unusual had happened, and it was not till, I suppose, the symptoms of rapidly advancing peritonitis ensued, that he desisted from his meal." To meet this difficulty the latest Lee-Metford has a nose of soft metal projecting beyond the nickel jacket. When the soft metal comes in contact with any obstruction it "mushrooms" out, the less yielding metal behind becomes impacted, and the ball opens into a disk, causing a very cruel wound. These modern projectiles are spoken of as "expanding" bullets. The celebrated "Dum-Dum" bullets are the latest form and derive their name from the government manufactory in India where they are made. They resemble a slate pencil and are a modification of the latest Lee-Metford, but the nickel shield is thinner and the soft nose is flat, and does not project so far beyond the nickel envelope. On impact this bullet collapses "like a concertina," and inflicts a ghastly wound. The extremest example of the expanding principle is to be seen in the "Webley's patent man-stopping bullet" which is cupped at each end. On entering the body the front acts like a wadding punch, cutting a clean circular hole which does not close up like those caused by other bullets. Expansion commences at once, the bullet assumes a shape something like "a lady's sun bonnet, or an antiquated top hat," and after travelling six inches it produces a jagged hole four inches in diameter. In many of the cases met with by Mr. Davis in the Græco-Turkish War it was impossible to tell by the appearance of the wounds which was the hole of entry and which of exit, the ball having passed straight through the part struck, leaving a tiny bluish-black hole at each end.

### The Physician's "Office."

NOTWITHSTANDING all that we hear about the English and the Americans being the same in race and language, in habits, customs, and all the rest, there is a difference, and this shows itself in little points, and sometimes where we would least expect it. One of these points is the doctor's consulting-room or "office." The consulting-room of the English physician—at any rate, of those physicians who do not practice specialities which require all sorts of wonderful apparatus—tends to assimilate itself to the library or the morning-room of a gentleman's house. In America, however, the tendency is to separate the office from the home, and in the office to make no inconsiderable display of the signs of one's calling. "One thing at a time" seems to be the motto over there; and when in the physician's office one can make no mistake as to one's locality. Even in offices, however, there are grades. Just as in England we see the consulting-room, the "surgery" of the general practitioner, or the "open surgery" under the red lamp; so in America the office may mean a gorgeously appointed apartment, bright with nickel-plated instruments and polished walnut furniture, or it may mean the barest of bare rooms. A short article in the *Charlotte Medical Journal*, entitled "How to Conduct your Office," is instructive and interesting, as showing some little peculiarities of medical life in the Carolinas. After criticising certain features common in such rooms, and pointing out that patients do not like dust and dirt, or carpets full of holes and ceilings lined with cobwebs, the writer says "the physician gives entirely too little thought to his office," and goes on to advise what should be done. He says, "Select for your office the best corner in the city (not at your home); take an upstairs room, furnish it as you would a parlour; then hire some one to clean and keep it in order, if you cannot, or will not, yourself." Having fixed up his office in this way, the physician, it is said, will not find it so much of a task to stay there when business is dull. "In fact, he will stay there and study, or be visited by his friends, instead of expectorating tobacco juice on the grocery floor, or swapping lies over the bar at the nearest groggery." A few other side lights on professional life and status are thrown in, and finally the very judicious advice is given that "a successful physician must office in his office, if he expects it to pay." Clearly, however, there is one link between physicians all the wide world over—they must always be at hand, and always be at the disposal of the public if they would get on. It is hard, but it is true; so let us make ourselves comfortable in our "offices."

